# Oral, Tongue-Coating Microbiota, and Metabolic Disorders: A Novel Area of Interactive Research

**DOI:** 10.3389/fcvm.2021.730203

**Published:** 2021-08-20

**Authors:** Yiwen Li, Jing Cui, Yanfei Liu, Keji Chen, Luqi Huang, Yue Liu

**Affiliations:** ^1^National Clinical Research Center for Traditional Chinese Medicine Cardiology, Xiyuan Hospital, China Academy of Chinese Medical Sciences, Beijing, China; ^2^The Second Department of Geriatrics, Xiyuan Hospital, China Academy of Chinese Medical Sciences, Beijing, China; ^3^China Academy of Chinese Medical Sciences, Beijing, China

**Keywords:** tongue coating microbiota, metabolic disorders, gut microbiota, mechanisms, oral microbiota

## Abstract

Interactions between colonizing microbiota and the host have been fully confirmed, among which the tongue-coating microbiota have a moderate rate of renewal and disease sensitivity and are easily obtained, making them an ideal research subject. Oral microbiota disorders are related to diabetes, obesity, cardiovascular disease, cancer, and other systemic diseases. As an important part of the oral cavity, tongue-coating microbiota can promote gastritis and digestive system tumors, affecting the occurrence and development of multiple chronic diseases. Common risk factors include diet, age, and immune status, among others. Metabolic regulatory mechanisms may be similar between the tongue and gut microbiota. Tongue-coating microbiota can be transferred to the respiratory or digestive tract and create a new balance with local microorganisms, together with the host epithelial cells forming a biological barrier. This barrier is involved in the production and circulation of nitric oxide (NO) and the function of taste receptors, forming the oral-gut-brain axis (similar to the gut-brain axis). At present, the disease model and mechanism of tongue-coating microbiota affecting metabolism have not been widely studied, but they have tremendous potential.

## Introduction

Microbial–host interactions closely influence human health status (1, 2). Microorganisms colonizing the human body can participate in the synthesis and metabolism of vitamins, proteins, and lipids, promote immunity, maintain the local ecological balance in organs, degrade nutrients, provide energy to the host (1, 3, 4) and have an important impact on host metabolic processes. In contrast, oral microbiota have gradually gained importance as easily detectable colonizing microorganisms, and research has shifted from oral diseases to a broader perspective, of which tongue-coating microbiota are an important part. Tongue-coating diagnosis is a pivotal aspect of traditional Chinese medicine (TCM). The TCM theory suggests that the tongue is fumigated by “stomach qi,” which indicates differences in disease etiology and disease status (5) and is widely used in clinical practice (6, 7).

Oral microbiota are not identical to tongue-coating microbiota. The oral cavity contains several habitats, and microbiota are distributed in the tongue coating, saliva, teeth, buccal mucosa (cheek), soft and hard palate, gingival sulcus, tonsils, throat, and lips (8, 9). Factors influencing the formation of oral microbiota include temperature, humidity, saliva volume, pH, oxygen, and the rate of local mucosal shedding (10, 11). Therefore, there is specificity in the microbiota of different loci. For example, there are significant differences in species and abundance between the tongue coating-derived conjugates of *Veillonella* and *Streptococcus* and dental plaque-derived complexes of *Veillonella* and *Streptococcus* (12). The tongue coating microbiota has stability with a moderate rate of shedding of biofilms formed by tongue epithelial cells and microbiota, making it a good site for study. In contrast, dental plaque-predictive sensitivity to disease may be inferior to that of tongue coating (13, 14). A higher rate of dental plaque shedding makes it less stable (14). Oral epithelial cells are renewed every 2.7 h (15), and rapid biofilm shedding affects the stability of the test results. The tongue dorsum is rich in filiform papilla, fungiform papillae, one row of annular papillae, and foliate papillae. It has a high diversity of bacterial communities, whereas, the non-keratinized epithelium at the base of the tongue can rapidly absorb small molecules and interact with the host. The proximity of the tongue to the tonsils allows compounds shed from epithelial cells and tongue-coating microbiota to be transported into the respiratory and digestive tracts. These characteristics make tongue-coating microbiota more likely to achieve oral-gut microbiota translocation and have broader metabolic effects.

Tongue-coating microbiota have also been associated with chronic systemic diseases, in which nutrients and metabolic disorders occur, such as gastritis and diabetes (16, 17) and different types of cancer (18–20). Due to its association with chronic non-oral diseases, tongue-coating microbiota are expected to serve as a potential markers for metabolic homeostasis and may be used as a future diagnostic tool. A metabolic disorder is an imbalance between uncoordinated digestion and absorption of substances in all diseases. Research directions in tongue-coating microbiota, its relationship with metabolic diseases, and its role in metabolism, are worth exploring.

## Overview of Tongue Coating Microbiota

### The Oral Microbiology Database

The National Institutes of Health Common Fund Human Microbiome Project (https://commonfund.nih.gov/hmp) (21) was established in 2007 and has previously examined the microbiota from nine oral cavity sites, including the tongue (22). Oral microbiota are relatively stable at the phylum level, but inter-host microbiota variation is high at both the species and strain levels (23). While published articles are available online (24), no tests on tongue-coating microbiota have been reported. Additional sequence analysis websites or databases (CORE et al.) have been developed based on the database (25, 26). Comparing high-throughput epidemiologic investigations,16S rRNA gene sequencing and the Human Oral Microbe Identification Microarray (HOMIM) provide similar sequencing detection sensitivity (27).

The Human Oral Microbiome Database (HOMD, http://www.homd.org/) was established in 2007 and is the first website that provides tools to describe human oral microorganisms systematically. Currently, 775 microorganisms are included online, of which 445 are from the oral cavity. Furthermore, 57% of the microorganisms were formally named, 13% were unnamed but cultured, and 30% were uncultured. A basic local alignment search tool (BLAST) (28) is available to search for genes and their annotations and is linked to JBrowse (29) to obtain information on the sequence of genes and other relevant information. The site allows searching for microbial strains by species taxonomic ID, genus, species, habitats, and nomenclatural status and provides a complete biotaxonomy list. However, currently, the colonization site is only localized to the oral cavity, and no detailed information is provided for tongue-coating-associated microbiota.

### Common Microbiota in Tongue Coating

The tongue-coating microbiota are structurally complex and contain not only monolayers of sparsely colonized bacteria but also equally free bacteria, bacteria on squamous epithelial cells, and structurally complex bacterial entities (consortia) (30). This shows that the epithelial cells of the tongue dorsum are a mixture of rapidly shed, sparsely colonized cells, and long-lived structures where more substantial biofilms can form. The microbiota and tongue papillae form a wide range of interspecies interactions that can be specifically classified as synergistic, signaling, or antagonistic (31, 32). Different species and genera of bacteria may have the same metabolic function, and the functional redundancy is widespread (30). Diversity and appropriate redundancy allow for greater stability (33) and metabolic efficiency (34). Therefore, the normal tongue coating microbiota diversity is higher than that of diseased individuals (20); however, microbiota abundance may be greater (35) in diseased than in healthy populations.

**Figure 1:**
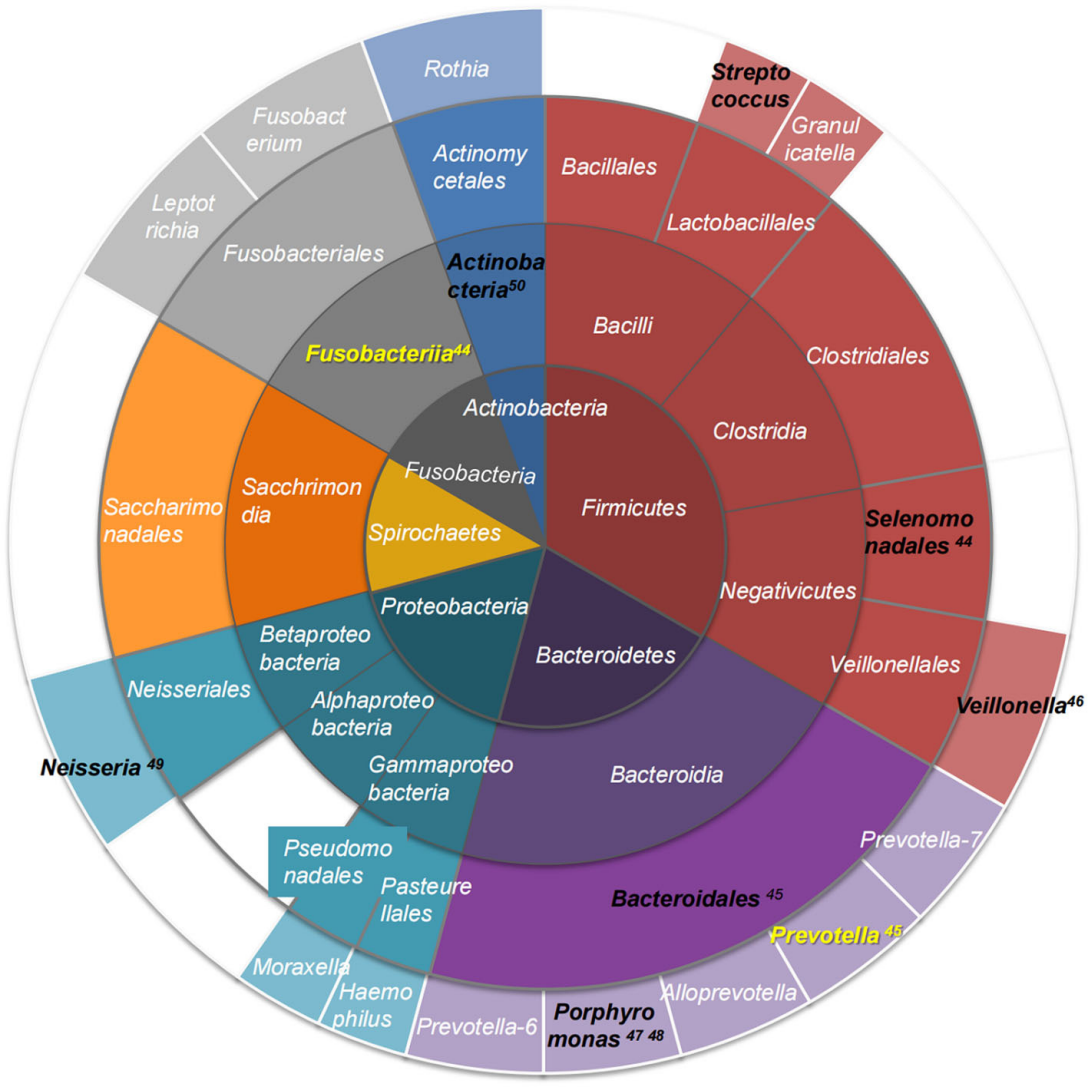
The dominant tongue coating microbiota at each taxonomic level. Different phyla are color-coded; layers from the inside to outside are phylum-class-order-genus. Black text indicates microbiota whose abundance is increased in metabolic diseases, and yellow text shows microbiota whose abundance is decreased in metabolic diseases (44–50).

Tongue-coating microbiota are reported more consistently at the phylum level, but with contradicting literature on the species level, which may be related to sampling methods, inclusion criteria, ethnicity, and region (14, 20, 36–39). The results of 16S rRNA analysis showed that tongue-coating colonizing microbiota in healthy humans at the phylum level included the following: *Firmicutes, Bacteroidetes, Proteobacteria, Actinobacteria, Spirochaetes, Fusobacteria*, and *Synergistetes* (27, 38, 40, 41), and the abundance of the top three microbiota was consistent with the overall distribution of the oral cavity. However, the proportions of *Actinobacteria* and *Spirochaetes* were higher than those of the oral cavity (42). The dominant tongue microbiota at each taxonomic level is shown in Figure 1 (14, 20, 43–50).

Common harmful oral bacteria can damage immunity and cause chronic inflammation (51, 52). Changes in the abundance of *Enterococcus sp*. and *Lactobacillus sp*. lead to the expansion of Th17 cells and the accumulation of IL-6 and IL-23 (53). Periodontitis not only originates from the gingival microbiota but is also affected by tongue-coating microbiota (14, 54). *Porphyromonas spp*. have been shown to induce atherosclerosis (55, 56) and diabetes (55). In obese individuals, tongue-coating *Bacteroidales* (order), *Enterococcus* (genus), and *Staphylococcus* (genus) were elevated, and *Prevotella* (genus) and *Butyricoccus* (genus) were decreased (50). In rheumatoid arthritis (RA) patients and at-risk individuals, tongue-coating *Prevotella* and *Veillonella* were higher in relative abundance than populations in healthy controls (13). RA patients present with an increased relative abundance of pro-inflammatory species.

Studies on oral probiotics have found that probiotic products containing *Lactobacillus* improve oral health status and may improve disease symptoms (57), such as fatty liver and cancer (53). Therefore, tongue epithelial cells have a barrier function (58) and are involved in taste production (44, 59), the oral-gut axis (60, 61), and nitric oxide (NO) cycling (62, 63). Thus, the role of tongue-coating probiotics in the host can be explored in several ways. Among nitrate-reducing bacteria, *Veillonella, Actinomyces, Haemophilus*, and *Neisseria* are highly abundant in the tongue coating and are potential probiotics (64).

### Tongue Coating Microbiota and Intestinal Microbiota

Intestinal microbiota are more extensively and intensively studied than tongue-coating microbiota (65, 66), and their relationships and whether the two have similar metabolic mechanisms deserve further exploration. Both tongue-coating microbiota and gut microbiota are associated with metabolic status (67), immune status, age (41), sex, genetic factors, environmental factors (68), antibiotic use, infant feeding status (69), and probiotic and prebiotic administration closely related to diet (70, 71). Colonization by intestinal and tongue-coating microbiota are dominated by anaerobic bacteria (69). The unweighted intestinal microbiota are separated from the oral microbiota compartment, indicating a large difference in the oral gut microbiota (72). Some can undergo microbiota displacement from the oral to the gut (37) or otherwise interact with each other. There are dozens of genera shared by the tongue coating and intestinal microbiota (72), such as *Lactococcus, Bilophila*, and *Akkermansia*. Oral and intestinal microbiota share nearly 45% homology but differ in their abundance.

**Figure 2:**
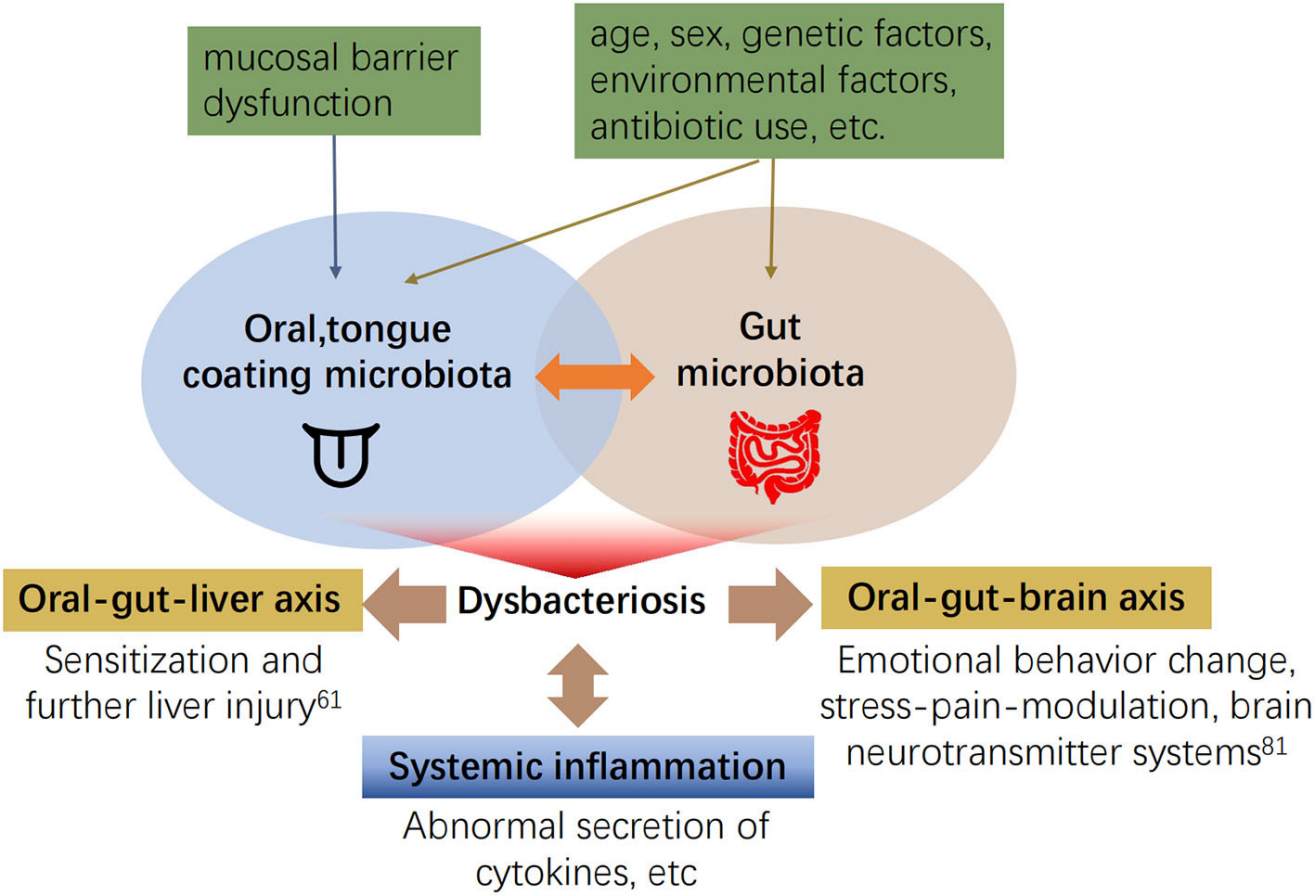
The interaction between tongue coating and gut microbiota and its effects on other systems (60, 61, 81).

Numerous studies have confirmed the strong relationship between gut microbiota and oral microbiota dysbiosis and systemic disease. However, the association between tongue coating microbiota and gut microbiota remains elusive. First, the tongue coating microbiota can translocate to the gut and lead to fluctuations in the gut microbiota. Recently, it was found that the abundance of 14 taxa has increased in tongue coating samples and stool samples from older adults (72), i.e., microbiota from other parts of the host may migrate to the oral cavity. The mechanism could be a decline in gastrointestinal function, or decreased gastric and bile acid secretion in the elderly. Oral microbiota are not inactivated and reach the intestine, invasion of gingival or tongue tissue by tongue coating microbiota, the impaired mucosal barrier function of the tongue epithelium, decreased levels of ligand proteins in the tongue (73), or Fap2-mediation (74) blood diffusion to reach the intestine (75). Second, changes in abundance in tongue coating microbiota are consistent with intestinal microbiota in disease, and both abundances are altered during the activation of immune receptors or abnormal hormone levels (76–78). In patients with autoimmune liver disease, *Veillonella spp*. are increased, and positively correlated in the oral and intestinal tracts (46). The tongue and intestine are involved in digestion through reflex stimulation of the gastric system, pancreas, liver, and gallbladder (79). Microbiota can also influence metabolism by interacting with taste receptors on the tongue and intestine (80), which may be related to the specific effector mechanism of the oral-gut axis (see Figure 2).

There are differences in the metabolic effects of gut and tongue coating microbiota. The production and oxidation of intestinal microbiotic metabolites, such as short-chain fatty acids (SCAFs) and branched-chain fatty acids, regulate energy expenditure (82). Gut microbiota also participate in the regulation of bile acid metabolism and the TMAO pathway. At present, the effects of tongue coating on related mechanisms have not been identified. The main metabolic process used by oral microorganisms is anaerobic respiration, whereas, microbial fermentation is the main process in the intestine (83). Therefore, the mechanisms involved in developing chronic systemic diseases by tongue coating microbiota differ from those of the intestinal microbiota.

### Tongue Coating Microbiota and Metabolic Status

The metabolites of tongue coating microbiota and chronic inflammation mutually promote each other. Tongue coating microbiota dysbiosis is directly associated with the development of periodontitis (14) and oral mucosal disease (84). Dental caries and periodontitis cause chronic infection, increase arterial inflammation and are high-risk factors for diabetes and cardiovascular disease (85–87). *Porphyromonas gingivalis* and *Fusobacterium nucleatum* have been shown to exacerbate chronic inflammation. C-reactive protein levels are elevated, and inflammatory responses are more severe in those with oral microbiota dysbiosis and are directly proportional to low-density lipoprotein (LDL) levels and the carotid intima-media area (60). The bacterial metabolites lipopolysaccharide (LPS) and IgG correspond to microbiota, and their activation of neutrophils can be detected in the blood of atherosclerosis patients (88), suggesting that the oral microbiota accelerates disease progression through endotoxin-activated immunity. Matrix-degrading metalloproteinases (MMPs) are important inflammatory mediators in cardiovascular disease and play a key role in the rupturing of atherosclerotic plaques (89), destruction of periodontal connective tissue, and exacerbating oral microbiota dysbiosis. Patients with myocardial infarction and periodontitis have elevated salivary MMPs and reduced tissue metalloproteinase inhibitor-1 levels, which may be related to oral microbiota metabolism (90). Periodontal microbiota causes widespread chronic inflammation and insulin resistance, exacerbating both the incidence and progression of Type 2 diabetes (91). Patients with periodontitis caused by chronic microbiota dysbiosis present with elevated serum LDL levels, decreased HDL levels, and elevated triglycerides, while periodontal treatment improves dyslipidemia and reduces total cholesterol and serum LDL levels. The pathogenesis of atherosclerosis is associated with oral microbiota, and poor oral hygiene can cause increased levels of fibrinogen and cell adhesion proteins (90). Endothelial dysfunction caused by oral microbiota leads to increased blood pressure (64).

Chronic diseases can also remodel the microbiota. In a mouse model, diabetes increased IL-17 expression, and chronic inflammation led to changes in the abundance of oral microbiota. Transplantation of altered oral microbiota to germ-free mice resulted in increased susceptibility to diabetes (92). Diabetes can cause changes in the oral microbiota (93). Comparison of oral microbiota between Type 2 diabetics and non-diabetics revealed *Neisseria spp., Fusobacterium, Veillonella*, and *Streptococcus spp*. increased.

The above studies on oral microbiota suggest a close relationship between tongue coating microbiota and chronic inflammatory and systemic diseases, especially metabolic diseases. Tongue coating microbiota has been confirmed as a potential biomarker for gastritis (20) and have predictive value for gastric cancer (20) and pancreatic head cancer (94). The correlation of tongue coating microbiota with greater metabolism is worth exploring.

### Mechanisms by Which Tongue Coating Microbiota Affects Metabolism

**Figure 3:**
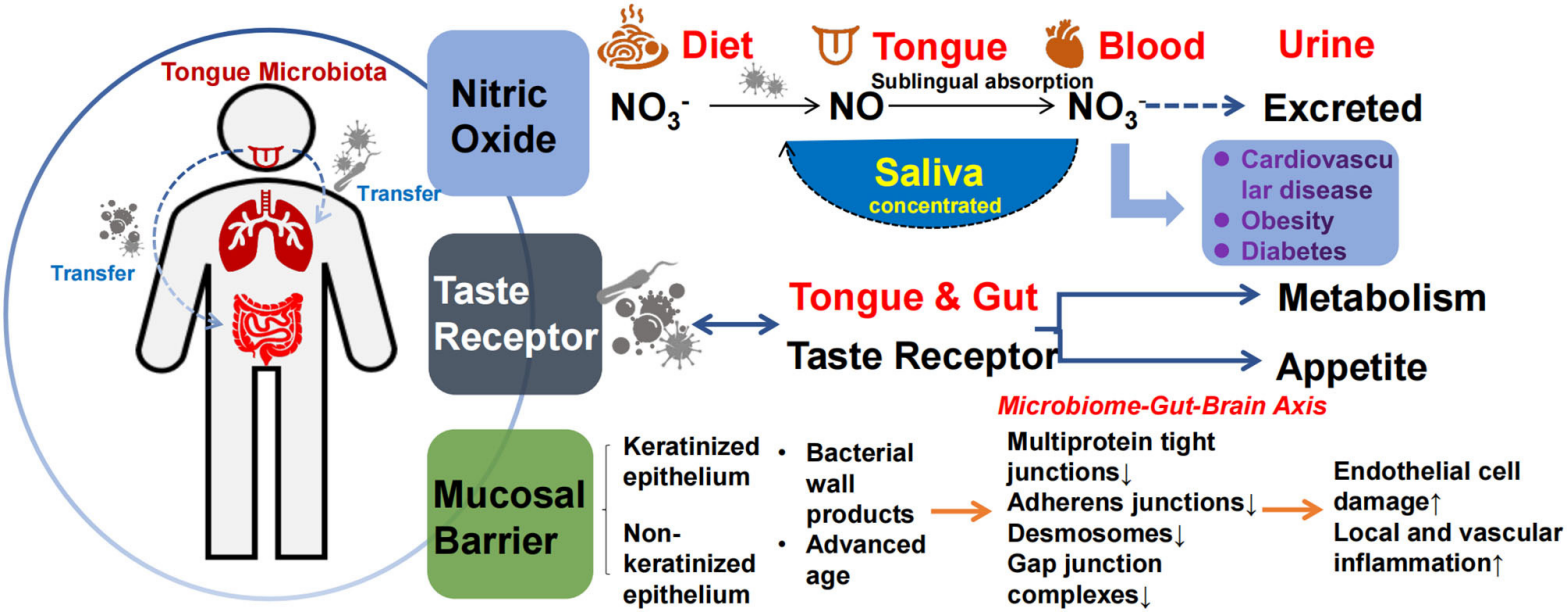
Mechanisms of tongue coating microbiota involved in metabolism. The tongue coating microbiota transfers or co-presents with other parts, including the respiratory tract and the gut (left). These three pathways may be involved in metabolism: (1) The production of nitric oxide (2) Receptor function (3) The formation of consortia with oral epithelial cells and entering the blood when the epithelial barrier is impaired (right).

The mechanism by which the tongue coating microbiota affects metabolism may be similar to intestinal microbiota, namely the biological barrier effect, involvement in the nitric oxide (NO) cycle, and taste production (45, 59). Metabolism in the oral cavity produces various antimicrobial compounds and enzymes, such as lysozyme, amylase, immunoglobulins, and epithelial cell shedding, which influence the establishment and renewal of the tongue coating microbiota. In turn, the tongue coating microbiota produces different metabolites and endotoxins, forming a complex barrier between the microbiota and the local environment of the tongue. Oral microbiota dysbiosis and cell wall production of endotoxins (e.g., LPS) promote inflammation and tissue destruction, and disruption of epithelial integrity further aggravates the penetration of oral microbiota into the oral epithelium and connective tissue (95). Studies have shown that microbiota, such as *Porphyromonas gingivalis*, can downregulate ligand protein expression and disrupt the oral mucosal barrier (96). On the other hand, the main sources of nutrients for microorganisms are saliva, host-consumed food, and various by-products produced between species, which support the growth and reproduction of microbiota, forming a biological barrier that competitively inhibits opportunistic pathogenic bacteria (see Figure 3).

Tongue coating microbiota play an important role in exogenous NO production and uptake, regulating host NO homeostasis. As a gaseous signaling molecule, NO is bound to human proteins *via* S-nitrosylation and plays an important role in a variety of physiological processes, including the regulation of vascular tone, nerve transmission, mitochondrial respiration, and skeletal muscle systolic function, thereby alleviating the development of diseases such as diabetes, hypertension, and coronary heart disease (97, 98). There are endogenous and exogenous pathways for NO production, and the microbiota-nitrate-nitrite-NO pathway serves as the main source of exogenous NO (99). NO production is non-strain-specific, and relevant microbiota–host interactions exist in the tongue and gut. Some microbiota can convert dietary nitrate to nitrite and produce NO *via* nitrate reductase or react with endothelial and plasma proteins to form S-nitrosothiol (SNO). Nitrite, NO, and SNO have activated soluble guanylate cyclase (sGC) and increased cGMP levels in tissues. The nitric oxide synthase (NOS)-NO pathway improves vascular tone through cGMP/PKG and cellular signals that stimulate smooth muscle relaxation (64, 94, 97). *Veillonella, Actinomyces, Prevotella, Neisseria*, and *Haemophilus* are among the most abundant NO-producing tongue coating microorganisms (99). NO in the tongue crypt can diffuse directly into the circulation through the highly vascularized tissues of the tongue, and regular tongue coating removal increases nitrate reduction to produce more NO (100) while reducing the production of sulfur-containing compounds to reduce bad breath. Microbiota also regulate the host gene expression profile by binding to various host proteins. For example, binding to argonaute protein inhibits miRNA activity to regulate host development (101). *In vitro*, excessive NO production can lead to developmental deformities in the host (101). In humans, dietary nitrate lowers blood pressure in healthy populations (102, 103). In the absence of any dietary changes, the use of mouthwash with sterilizing effects can disrupt oral microbiota, thereby reducing oral and plasma nitrite levels in healthy populations, and is associated with a sustained increase in systolic and diastolic blood pressure (102, 103). Thus, NO production by the oral microbiota in healthy humans has positive implications for vascular endothelial homeostasis (see Figure 3).

In contrast, the chemosensory system in mammals is regulated by bacterial metabolites, and tongue coating microbiota may also be involved in taste formation, affecting eating and metabolism (104). In traditional Chinese medicine, the relationship between the five tastes and the internal organs was proposed as early as the period of the “Inner Canon of the Yellow Emperor: sour taste enters the liver, astringent taste enters the lung, and bitter taste enters the heart.” These observations have been clinically corroborated in long-term clinical practice (105). The taste system mainly includes carbohydrate, amino acid, fat receptors (58), and bitter receptors. Through the above feeding sensations, the function of the tongue establishes a close relationship with the metabolic system. Obese children have significantly lower taste discrimination and fewer fungiform papillae, accompanied by reduced α-diversity of tongue coating microbiota. In healthy subjects, tongue coating microbiota was associated with taste function, thereby affecting dietary habits, such as preference for salty baked products, saturated fat-rich products. Beverages are consumed more frequently by those who are insensitive to salty flavors than by those who are sensitive.

Likewise, sweet foods are consumed more frequently by those who are insensitive to sweet flavors (106). *Prevotella* abundance is positively associated with vegetable intake, whereas, *Clostridia* abundance is associated with protein/fat-rich diets (106). A recent study of the human microbiome found that commensal bacteria have developed strategies to stimulate chemosensory receptors and trigger host cell function (104). Thus, tongue coating microbiota may impact metabolic systems through interactions with chemosensors on the tongue. Similar to the gut microbiota, several previous studies have elucidated feedback mechanisms in gut microbiota-microbiota products-intestinal epithelial cells-endocrine metabolic homeostasis (107, 108) and have inhibitory effects on gastrointestinal motility and appetite through GLP-1, CCK-, ghrelin-, and peptide tyrosine tyrosine (PYY)-labeled EECs in the human small intestine and colon (109). It can be suggested that tongue coating microbiota and their hosts share similar interaction mechanisms.

## Conclusions

Tongue coating microbiota, one of the important components of the oral microbiota, have high sampling stability. Disturbances in tongue coating microbiota have been shown to elevate various chronic inflammatory markers, as well as being closely linked to mechanisms such as local mucosal barriers, nitric oxide metabolism, and taste chemoreceptors. Although, there is a lack of studies of drug action on tongue-coating microbiota to treat disease, studies have shown that probiotics can modulate oral microbiota and improve health (110, 111). In contrast, frequent use of antibiotics or mouthwash adversely affects blood pressure (102, 103). Therefore, tongue coating microbiota are expected to become a new, easy, and non-invasive biological marker that can contribute to diagnostic and prognostic studies of chronic non-infectious diseases.

Research on tongue coating microbiota has not been extensively conducted, and the number of studies related to metabolic diseases and metabolic mechanisms is limited in the field of oral microbiota. Tongue coating microbiota and intestinal microbiota show some similarity in composition, as both are involved in food digestion. Based on studies on intestinal microbiota, tongue coating microbiota may also be involved in various metabolic mechanisms in the human body. The standard sampling procedure and research paradigm of tongue coating microbiota should be standardized, and multi-omics studies or tongue-gut clustering analysis should be performed in the future.

## Author Contributions

YueL contributed to the topic design, manuscript revision, and the decision to submit for publication. YiwL, JC, and YanL performed the literature retrieval, collation, analysis, and wrote the manuscript together. YiwL and JC were co-first authors. LH and KC revised the manuscript. All authors approved the final version of the manuscript.

## Conflict of Interest

The authors declare that the research was conducted in the absence of any commercial or financial relationships that could be construed as a potential conflict of interest.

## Publisher's Note

All claims expressed in this article are solely those of the authors and do not necessarily represent those of their affiliated organizations, or those of the publisher, the editors and the reviewers. Any product that may be evaluated in this article, or claim that may be made by its manufacturer, is not guaranteed or endorsed by the publisher.
